# *Phragmites australis* and *Argyrogramma albostriata* Suppress the Invasion of *Solidago canadensis* in China Under Future Climate Change

**DOI:** 10.1002/ece3.73573

**Published:** 2026-04-22

**Authors:** Jinghui Zhang, Xiaoying Xiao, Wei Huang, Yuxin Huo, Yuxin Zhang, Shujian Zhang, Xinyi Huang, Muhammad Umair Hassan, Yuxi Xue, Qitao Su, Yian Xiao

**Affiliations:** ^1^ Key Laboratory of Jiangxi Province for Biological Invasion and Biosecurity, School of Life Sciences Jingangshan University Ji'an Jiangxi China

**Keywords:** biological control, habitat distribution, habitat tracker, invasive plant control, MaxEnt model

## Abstract

Global warming has significantly accelerated the invasion process and expanded the distribution range of *Solidago canadensis.* It forms a dominant monoculture in multiple regions via wind‐dispersed seeds and rhizome clonal propagation, posing a serious threat to agricultural productivity and biodiversity. The native *Phragmites australis* suppresses its expansion through ecological niche competition, while the specialized predator *Argyrogramma albostriata* significantly reduces its population density through feeding. This study used the MaxEnt model, which offers advantages such as high predictive accuracy, simplicity of operation, and short computation time, making it widely applied in research related to climate change and species distribution. By incorporating diverse environmental variables including bioclimatic factors, it simulates the potential distribution patterns of these species under current (1970–2000) and future (2041–2060, 2081–2100, SSP126/SSP245/SSP585 scenarios). Results indicate that under future climate scenarios, the suitable habitat of *P. australis* may synchronously cover the potential distribution range of *S. canadensis* as the latter expands, providing a spatial foundation for ecological niche competition control. The habitat of the exhibits a “tracking effect” toward the core invasion zones of *S. canadensis* (e.g., East China, South China), supporting the potential for specialized feeding control. Moreover, the logic of habitat overlap between these species and *S. canadensis* remains intact despite climate warming. These findings elucidate the *S. canadensis* invasion dynamics under global warming, providing a theoretical groundwork for establishing a synergistic control system integrating native plant competition and natural enemy regulation.

## Graphical abstract

Global warming is driving the spread of *Solidago canadensis* and threatening the ecosystem and agriculture. The ecological niche competition of the native *Phragmites australis* and the exclusive feeding of the Argyrogramma albostriata have the potential for coordinated control. This study simulated the suitable zones of the three under different current and future climate scenarios through the MaxEnt model and found that the suitable zones of the two can simultaneously cover or track the core distribution areas of invasive species, providing key theoretical support for the construction of a “native plant competition – natural enemy regulation” coordinated prevention and control system.

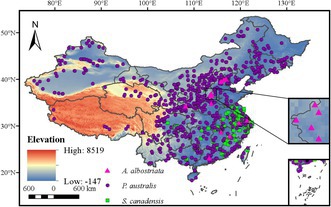

## 1 Introduction

Globally, invasive alien plants represent a major environmental threat to ecosystem stability and sustainability. By outcompeting native species, they displace local flora, disrupt fundamental ecological processes, and contribute to biodiversity loss, ultimately leading to substantial economic costs (Díaz et al. 2019). Asia is a hotspot for biological invasions, where intensive agriculture, rapid urbanization, and frequent international trade collectively accelerate the spread and ecological influence of invasive species in this region (Van Kleunen et al. 2015). This trend is notably evident in China, where invasive plants severely disrupt both the structure and function of native ecosystems (Yan et al. 2001) and incur substantial socioeconomic costs (Xu et al. 2006). Although this earlier estimate represents a milestone assessment, current actual losses are likely far higher due to worsening invasion pressure. Of these threats, invasive plants constitute a primary driver of ecological degradation. They directly destabilize ecosystems by outcompeting native species and establishing monospecific stands (Vilà et al. 2011). Extensive research has investigated the dispersal mechanisms and management strategies of invasive plants (Seastedt 2015). Current management strategies primarily include physical, chemical, and biological control. Physical control tends to be costly and inefficient, while chemical control, despite its economically effectiveness, often leads to environmental pollution (Pimentel 2005). Biological control provides a more efficient, environmentally sustainable, and safer management strategy. It functions through mechanisms such as competing with pathogens for resources, secreting secondary metabolites, and inducing systemic resistance in plants (Compant et al. 2005), these processes enhance plant immunity and vigor, contributing to sustainable disease management. Owing to its advantages—including environmental safety, high target specificity, and sustainability (Hajek and Eilenberg 2018)—biological control is now widely incorporated into integrated pest and weed management programs (Kato‐Noguchi 2023) However, its implementation requires careful risk assessment to avoid non‐target effects and unintended ecological impacts.

*Solidago canadensis* L., a perennial herb native to North America, is a member of the genus Solidago in the Asteraceae family. It has become a widespread invasive species on multiple continents, such as Europe, Asia, and Australia (Tian et al. 2023). Its growth form is characterized by annual aerial stems and a perennial underground rhizome system (Hao et al. 2010). Due to its high reproductive output and wide adaptability, *S. canadensis* is considered a classic example of a globally invasive plant (Su et al. 2025). *S. canadensis* competes intensely with native plants for resources, with light capture being a key component of its competitive success. The species casts dense shade due to its tall growth and crowded canopy, which reduces light availability for understory plants and suppresses their photosynthesis (Baranová et al. 2022). Concurrently, it achieves high light‐use efficiency via greater leaf area and elevated chlorophyll content, enabling rapid biomass accumulation under favorable light conditions (Kama et al. 2023), this combination allows *S. canadensis* to dominate light resources, securing its competitive dominance following invasion. Introduced to China around the mid‐20th century, it has spread aggressively across eastern regions and is now a major invasive threat (Dong et al. 2015). A special survey organized by the Jiangsu Provincial Department of Natural Resources (https://zrzy.jiangsu.gov.cn/) revealed that as of late November 2024, the invasive area of *S. canadensis* across the province reached approximately 5300 ha. However, most existing have focused solely on predicting the potential distribution of *S. canadensis* using climatic variables. There is a notable lack of integrated modeling that combines its distribution with that of its key native competitor ( *P. australis* ) and specific natural enemy ( *A. albostriata* ), which limits our ability to design spatially explicit, synergistic biocontrol strategies.

*Phragmites australis* (Cav.) Trin. ex Steud. is a rhizomatous grass with a pronounced capacity for clonal reproduction. Natural populations regenerate mainly through rhizome propagation (Oborny and Bartha 1995; Fér and Hroudova 2009), a strategy that frequently results in monodominant or codominant stands (Meyerson et al. 2000). As a dominant native wetland species, it exhibits strong competitive abilities for resources and releases allelopathic compounds (Li et al. 2011). Through allelopathy and intense competition for light, water, and nutrients, *P. australis* effectively suppresses the establishment and growth of *S. canadensis* , while also limiting the spread of this and other invasive species via niche competition (Zhang et al. 2014). By virtue of its high‐biomass growth pattern, it competitively absorbs water and nutrients. Its dense rhizome system forms a physical barrier layer that limits the establishment space of invasive plants, thereby weakening their ecological expansion capacity (Ryabov et al. 2010). Furthermore, a specific natural enemy of *S. canadensis* (Chen et al. 2016) and larvae directly inhibit the accumulation of photosynthetic products and the formation of propagules in the host by intensively grazing on leaves and inflorescences. Significant population regulatory capacities in the primary invasion sites (Pannuti et al. 2016). *P. australis* , as a competitor of *S. canadensis* , and *A. albostriata* , as a natural enemy of *S. canadensis* , both significantly suppress the growth of *S. canadensis* in their shared habitat. Therefore, by predicting the future suitable habitat distributions of *S. canadensis* , *P. australis* , and *A. albostriata* , coupling the ecological niche requirements of both plants and the predatory insect, a synergistic control system based on native plant competition and specific predator regulation can be established (Chen et al. 2025). This provides theoretical foundations and practical pathways for the scientific control and ecological management of this invasive species, holding key significance for developing governance strategies grounded in ecological niche competition and biological interactions.

Species Distribution Models (SDMs) can be used to identify the potential distribution areas of invasive species, thereby effectively supporting risk assessment and the optimization of prevention and control strategies. By employing SDM simulations to analyze the ecological hazards and risks associated with alien species invasions, these models provide quantitative evidence regarding invasion likelihood and potential pathways. This scientific foundation supports early warning systems and informed decision‐making for prevention and control measures (Yin et al. 2022). The maximum entropy model (MaxEnt 3.4.1) has become a benchmark tool in invasion ecology research due to its robustness to small sample sizes and high accuracy (Su et al. 2024). It is widely used in climate change responses, assessment of threatened species habitat conservation, and prediction of invasive plant spread areas (Liao et al. 2025).

Although nationwide analyses indicate that its suitable habitats are widely distributed across most parts of East, Central, and Southwest China (Li et al. 2017), however, existing studies have primarily focused on predicting its potential distribution, with limited exploration of how biotic and abiotic factors interact to shape its distribution patterns. Under global warming, a critical question remains: how can niche models be used to systematically evaluate the potential of a combined “native plant competition–specialized natural enemy” strategy to curb the invasion of *S. canadensis* ? To address this, we applied the MaxEnt to simulate the potential distributions of *S. canadensis* and its control agents across current and future climate scenarios, incorporating bioclimatic, topographic, and anthropogenic variables. This study aims to: the system simulated the potential distribution patterns of *S. canadensis* and its natural enemies under the current climate baseline and future scenarios. Therefore, this study aims to: (1) predict current and future suitable habitats for *S. canadensis* , *P. australis* , and *A. albostriata* in China; (2) assess spatial overlap and identify potential zones for synergistic control among the three species; (3) propose that areas of high distributional overlap among these species represent priority zones for implementing an integrated competition–predation biocontrol strategy.

## 2 Materials and Methods

### 2.1 Distribution Data of Species

**FIGURE 1:**
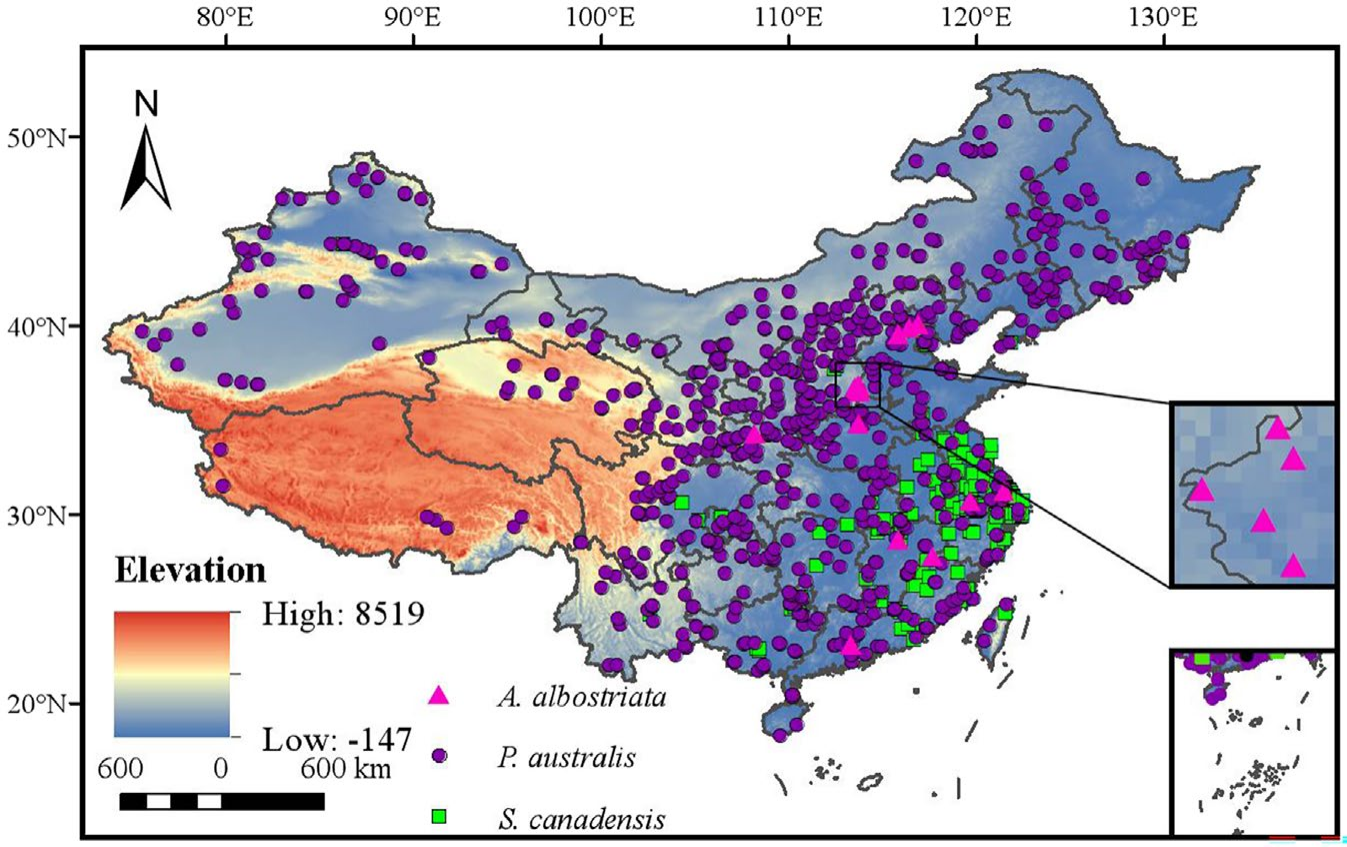
Distribution maps of *S. canadensis* , *P. australis* , and *A. albostriata* in China.

Distribution data for *S. canadensis* , *P. australis* , and *A. albostriata* were obtained through literature review and database retrieval. The primary sources included the Global Biodiversity Information Facility (GBIF; https://www.gbif.org/, accessed on 10 December 2024), the Chinese Virtual Herbarium (CVH; https://www.cvh.ac.cn/, accessed on 10 December 2024), the China Animal Scientific Database (http://www.zoology.csdb.cn, accessed on 10 December 2024), the Encyclopedia of Life (https://www.eol.org/zh‐CN, accessed on 10 December 2024), and published records from the Flora of China. To ensure data quality, occurrence records were filtered according to the following criteria: (1) retaining only records with precise geographic coordinates (spatial uncertainty < 5 km); (2) excluding duplicate records and those from obvious cultivation, introduction, or rearing sites; and (3) limiting the temporal range to records from 1970 onward to align with the baseline climate period. To mitigate the effects of sampling bias and spatial autocorrelation on species distribution models, we first removed all records lacking geographic coordinates. We then used ENMTools to spatially thin occurrence points with a 1 km distance threshold: Euclidean distances were calculated for all point pairs, and any pair with a distance < 1 km was treated as spatially duplicate, retaining only one record per cluster. The resulting spatially filtered dataset was exported as a CSV file and subsequently used as input for MaxEnt (Zhang et al. 2018). After screening, a total of 241 effective distribution points for *S. canadensis* , 692 for *P. australis* , and 16 for *A. albostriata* were obtained across China (Figure 1). The filtered distribution point data were then compiled into .csv files organized by species name and distribution point coordinates to facilitate MaxEnt model development and simulation‐based predictive analysis.

### 2.2 Environmental Variables

Environmental variables for modeling were obtained from multiple sources. Present (1970–2000) and future (2041–2060, 2081–2100) bioclimatic data (Bio1–Bio19, Table S1) at a spatial resolution of 2.5 arc min (~1 km) were downloaded from WorldClim version 2.1 (https://www.worldclim.org/) (Fick and Hijmans 2017). This dataset was selected for its high spatial resolution and the provision of long‐term climate normals that are pertinent to species physiological tolerances. Future projections were derived from the BCC‐CSM2‐MR global climate model under the CMIP6 (Coupled Model Intercomparison Project Phase 6) scenario framework (SSP126, SSP245, SSP585) (Eyring et al. 2016), which were statistically downscaled and bias‐corrected to the same 2.5 arc min resolution by WorldClim. Three topographic variables—elevation (Alt), aspect, and slope—were derived from Digital Elevation Models (DEMs) provided by the Computer Network Information Center of the Chinese Academy of Sciences (CAS) and the International Scientific Data website (http://www.gscloud.cn/), with a resolution of 25 m. Human Activity Intensity (HA) data, also known as the Global Human Impact Index, was sourced from SEDAC (https://ciesin.columbia.edu/sedac). The time periods selected are 2041–2060 and 2081–2100. All downloaded environmental raster layers were preprocessed in ArcGIS 10.8 to ensure spatial consistency and compatibility with MaxEnt. First, layers were projected to a unified coordinate system (WGS 1984) using the Project Raster tool. Second, all layers were resampled to a uniform spatial resolution of 25 m using the Resample tool (bilinear interpolation for continuous variables; nearest neighbor for categorical variables). Third, each layer was clipped to the extent of China's administrative boundary using the Extract by Mask tool, with a vector polygon of China's national border as the mask. Finally, the processed raster layers were converted to ASCII format using the Raster to ASCII tool, serving as the input format required by MaxEnt. These preprocessing steps ensured full spatial congruence among all environmental variables prior to modeling.

### 2.3 Maximum Entropy Model (MaxEnt) Simulation

The MaxEnt was employed to predict the potential habitat of *S. canadensis* . This modeling approach is particularly suitable for invasive species studies because it requires only species occurrence data and does not rely on difficult‐to‐obtain true absence records (Elith et al. 2011). The built‐in regularization procedure effectively prevents overfitting, enabling robust estimation of species environmental responses even with limited sample sizes. Furthermore, the continuous habitat suitability probabilities generated by MaxEnt allow for quantitative comparison of niche overlap and shifts among the invasive plant, its competitor, and the natural enemy (Merow et al. 2013), thereby providing a coherent analytical framework for evaluating the spatiotemporal feasibility of the synergistic control strategy. Model accuracy was assessed using the Area Under the Receiver Operating Characteristic Curve (AUC value). AUC values range from 0.5 to 1, with higher values indicating greater prediction reliability. The grading criteria are: 0.5–0.6 (prediction failure), 0.6–0.7 (poor), 0.7–0.8 (fair), 0.8–0.9 (good), 0.9–1.0 (excellent) (Liao et al. 2025). Imported the filtered distribution point data (*n* = 241) and 23 environmental variables of *S. canadensis* into MaxEnt software. The model was run 10 times with repeated Cross‐validation simulations (Phillips et al. 2006), across these 10 replicate runs, the percent contribution of each environmental variable was averaged to obtain a robust assessment of its relative importance generating predictions for potential suitable areas under both contemporary (1970–2000 baseline) and future scenarios (2040s/2080s/2100 s under SSP126/SSP245/SSP585). The model‐generated Logistic format raster data were extracted using ArcGIS 10.8, overlaid with vector boundaries of China's provincial administrative divisions, and compiled into a national‐scale potential distribution map.

Niche consistency among *S. canadensis, P. australis*, and *A. albostriata* species was tested using ENMTools (Warren et al. 2010). Within ENMTools version 1.1.2, distribution layers under current climatic conditions were generated with MaxEnt. These layers were used to calculate the observed values of Schoener's D (Schoener 1968) and Warren's I (Warren et al. 2008). The frequency distribution of expected values for these metrics was obtained by performing 100 iterations on pseudo‐replicate datasets. A non‐parametric Monte Carlo permutation test was applied to assess the statistical significance between the observed and expected values of the evaluation indices. The hypothesis of niche consistency was rejected when the actual I and D values were significantly lower than the expected values from the pseudo‐replicate datasets (*p* < 0.01), indicating that niche differentiation had occurred between the two species (Broennimann et al. 2012) (Petitpierre et al. 2012).

### 2.4 Suitable Distribution Zones

Based on the MaxEnt output results, the SDMToolbox toolkit was used to classify suitable habitats into three tiers: unsuitable areas (training set below the 10th percentile), suitable areas (training set between the 10th percentile and 0.66), and highly suitable areas (> 0.66). The proportion of suitable habitat area for each tier was ultimately calculated. The final MaxEnt model was constructed using the software's default settings for feature classes (Quadratic, Product, and Hinge; QPH) and regularization multiplier (1.5). This parameter combination is widely adopted in species distribution modeling as it provides a robust balance between model complexity and generalizability, effectively mitigating overfitting risks (Phillips et al. 2006). The Jackknife method was applied to quantify the contribution rates of environmental factors and their replacement significance values. Import the MaxEnt‐generated ASC result file into ArcGIS 10.8. Convert the ASC raster to GeoTIFF format and calculate area changes for *S. canadensis* under current and future scenarios (2040s/2080s/2100s SSP126/SSP245/SSP585).

## 3 Results

### 3.1 Model Accuracy Assessment

This study evaluated the model accuracy of *S. canadensis* using multiple iterations of ROC curve analysis with the MaxEnt model. Model performance was further assessed using the True Skill Statistic (TSS), calculated as TSS = Sensitivity + Specificity −1, where values range from −1 to +1, with higher values indicating better prediction. The mean AUC exceeded 0.96 (Table 1), significantly surpassing the 0.9 threshold for excellent performance, indicating highly reliable prediction results.

**TABLE 1:** The 23 environmental variables used for model prediction.

Species	AUC training	AUC test	MTSS
*Solidago canadensis*	0.990	0.990	0.306
*Phragmites communis*	0.966	0.963	0.346
*Argyrogramma albostriata*	0.991	0.997	0.406

**FIGURE 2:**
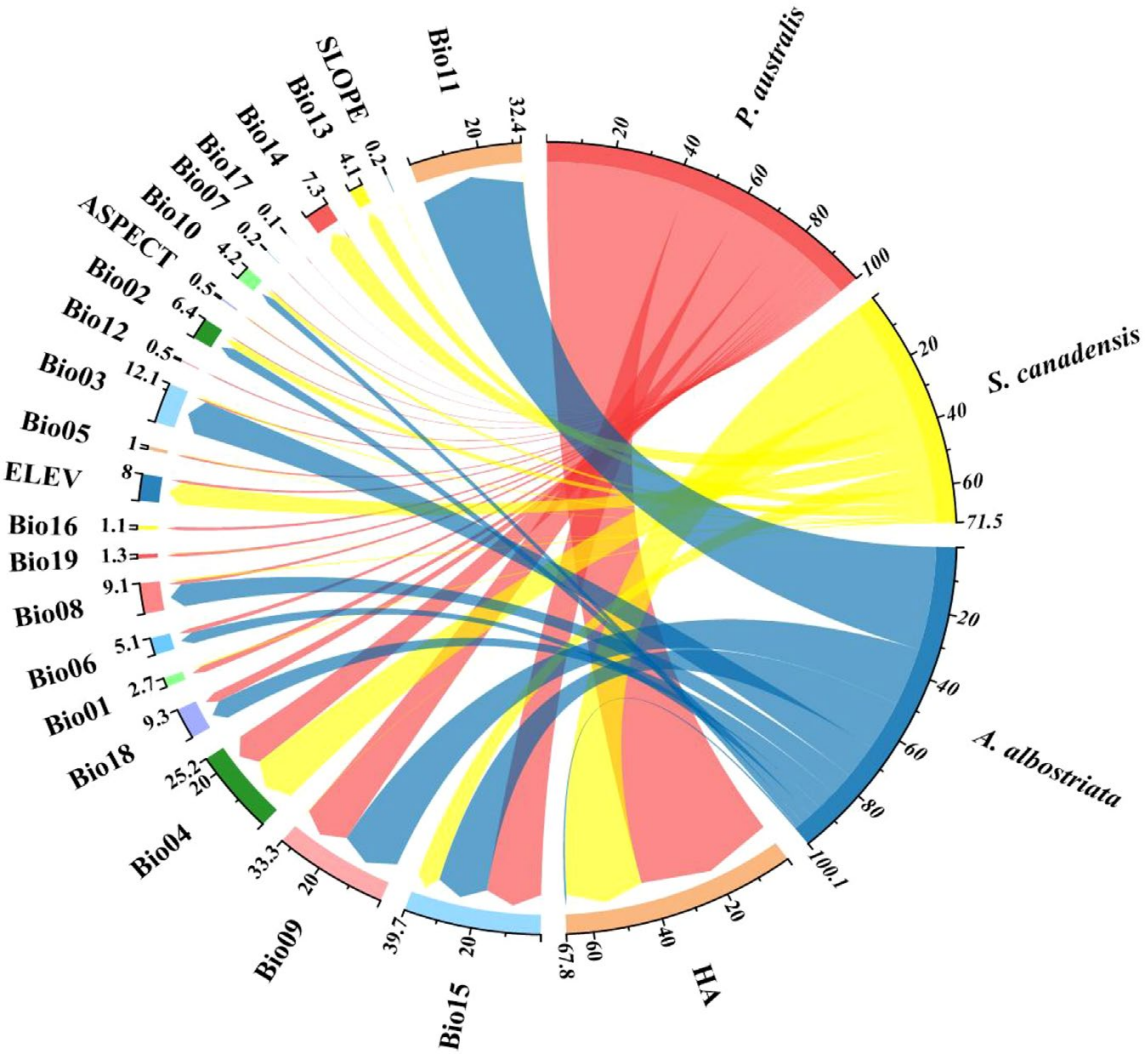
The percentage contributions of environmental factors for *S. canadensis* , *P. australis* , and *A. albostriata*.

**FIGURE 3:**
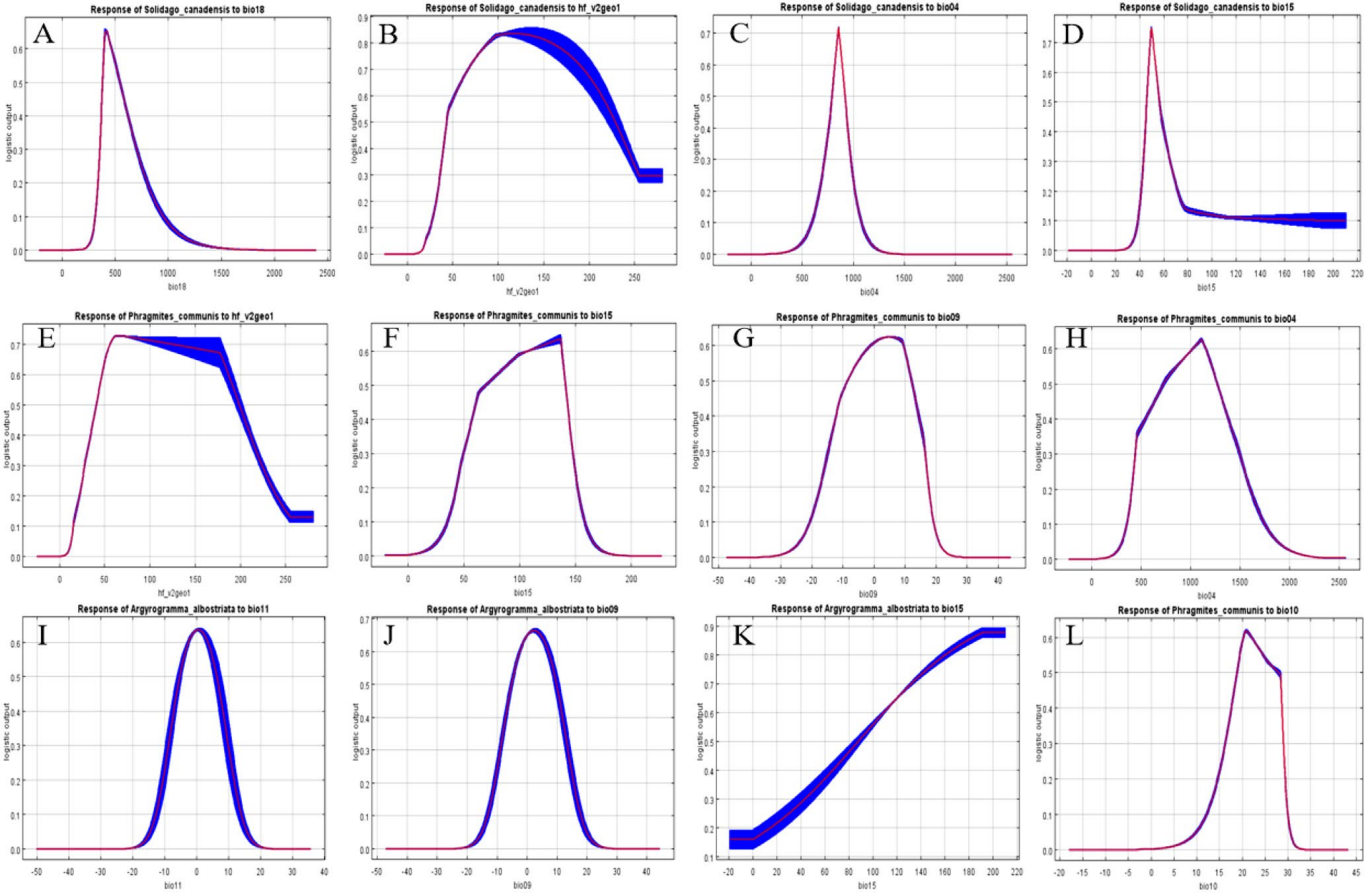
Response curves of key environmental factors for *S. canadensis* , *P. australis* , and *A. albostriata* (A–D: show the primary environmental factors affecting *S. canadensis* : Precipitation of warmest quarter, Human activity, Temperature seasonality, Precipitation seasonality; E–H: show the primary environmental factors affecting *P. australis* : Human activity, Precipitation seasonality, Mean temperature of driest quarter, Temperature seasonality; I–L: show the primary environmental factors affecting *A. albostriata* : Mean temperature of coldest quarter, Mean temperature of driest quarter, Precipitation seasonality, Mean temperature of warmest quarter).

When the model was run using the Jackknife method, the results indicated that among the 23 variables examined (Table 1), the MaxEnt model analysis determined the contribution percentages of 23 influencing factors (Figure 2). The four primary environmental factors affecting the distribution of the *S. canadensis* (Figure 3A–D) were Bio18 (Precipitation of warmest quarter), HA (Human activity), Bio04 (Temperature seasonality), and Bio15 (Precipitation seasonality). The four primary environmental factors influencing the distribution of *P. australis* (Figure 3E–H) were HA (Human activity), Bio15 (Precipitation seasonality), Bio09 (Mean temperature of driest quarter), and Bio04 (Temperature seasonality). The four primary environmental factors influencing the distribution of the *A. albostriata* (Figure 3I–L) are Bio11 (Mean temperature of coldest quarter), Bio09 (Mean temperature of driest quarter), Bio15 (Precipitation seasonality), and Bio10 (Mean temperature of warmest quarter).

When the habitat suitability probability of a species exceeds 0.5 (Figure 3), it indicates that *S. canadensis* , *P. australis* , and *A. albostriata* are suitable for growth in that environment. When the probability of *S. canadensis* occurrence exceeds 0.5, the precipitation of the warmest quarter ranges from approximately 350 to 550 mm, temperature seasonality is between 750 and 900, precipitation seasonality is 45 to 55, and human activity exceeds 40. When the probability of *P. australis* occurrence exceeds 0.5, Precipitation seasonality ranges from 70 to 140 mm, Mean temperature of driest quarter is −8°C to 12°C, Temperature seasonality spans 700 to 1250, and Human activity falls between 40 and 200. When the probability of *A. albostriata* occurrence exceeds 0.5, the mean temperature of the coldest quarter ranges from −5°C to 7°C, the mean temperature of the driest quarter ranges from −5°C to 10°C, precipitation seasonality exceeds 90 mm, and the maximum temperature of the warmest month ranges from 19°C to 28°C.

### 3.2 Distribution of *S. canadensis* , *P. australis* , and *A. albostriata* Under Current Climate

**FIGURE 4:**
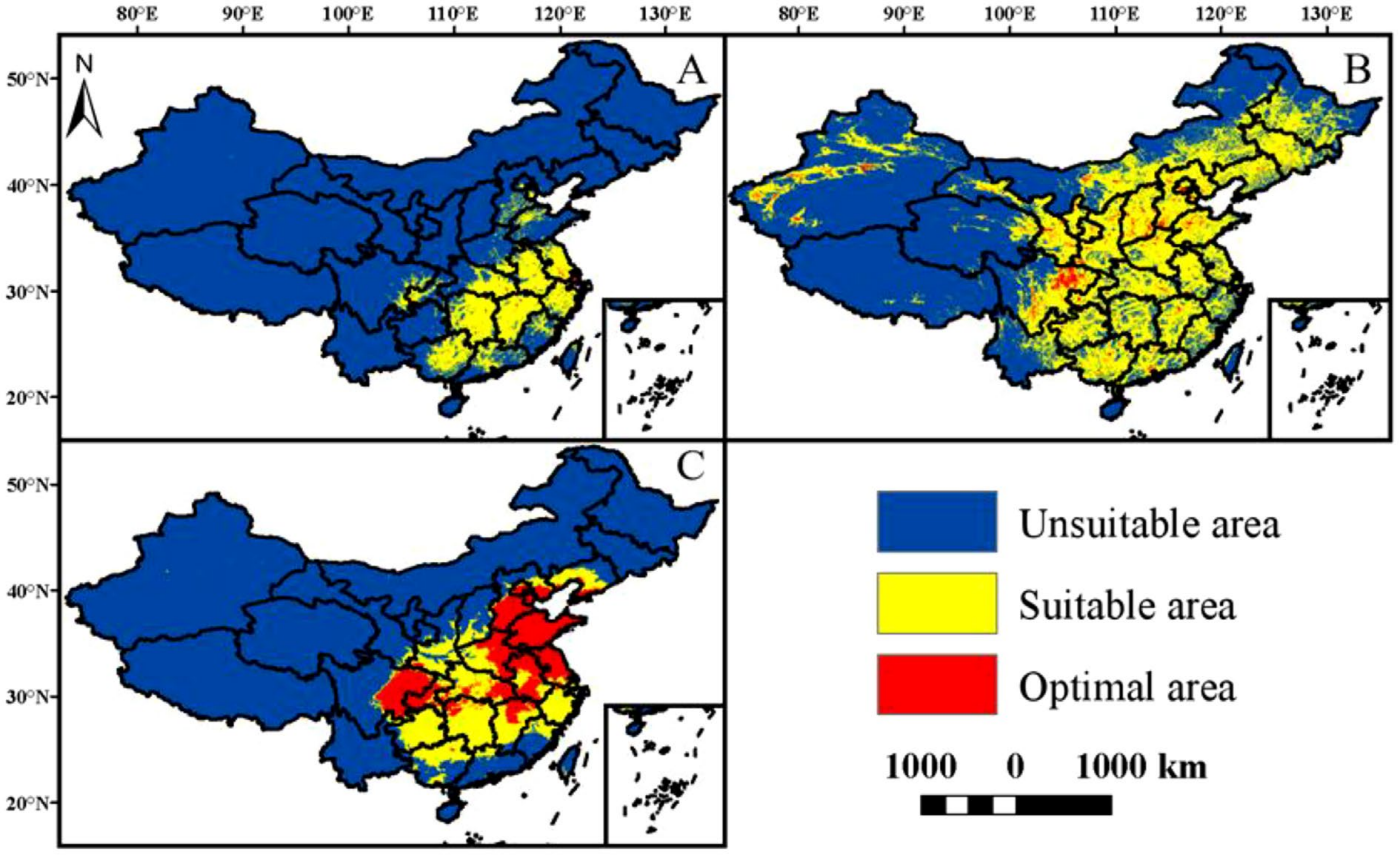
Current suitable habitat distributions of *S. canadensis* , *P. australis* , and *A. albostriata* (A: shows the suitable habitat distribution of *S. canadensis* ; B: shows the suitable habitat distribution of *P. australis* ; C: shows the suitable habitat distribution of *A. albostriata* ).

*S. canadensis* is widely distributed across central and eastern China, with its high‐suitability zones primarily concentrated in the middle and lower reaches of the Yangtze River plain (eastern Hubei, northern Hunan, northern Jiangxi, southern Anhui, and eastern Shanghai), covering a total area of 89.75 × 10^4^ km^2^. The distribution of suitable habitats for *P. australis* in China is widely distributed across the North China Plain, the Southwest Plateau, and the Northwest Oasis regions. Its high‐suitability zones are primarily concentrated in the middle and lower reaches of the Yangtze River: Dongting Lake (Hunan), Poyang Lake (Jiangxi), and Taihu Lake (Jiangsu) lakeside wetlands (continuous patch distribution); Northeast marshlands: Sanjiang Plain (Heilongjiang), Liaohe Delta (Liaoning); Coastal zones: Bohai Bay tidal flats (Tianjin‐Hebei), Pearl River Estuary tidal flats (Guangdong). Total area: 383.46 × 10^4^ km^2^. The distribution of the *A. albostriata* in China is shown in Figure 4C. *A. albostriata* is widely distributed in the southern North China Plain, the Yunnan‐Guizhou Plateau, and the lower Yangtze River region. Its high suitability zones are primarily concentrated in the middle Yangtze River region: the Jingzhou, Hubei‐Yueyang, Hunan corridor; Sichuan Basin: Chengdu Plain and surrounding hills; Southeast coastal region: Fujian Minjiang Estuary to Guangdong Pearl River Estuary, covering a total area of 212.72 × 10^4^ km^2^.

### 3.3 Potential Habitat Changes for *S. canadensis* , *P. australis* , and *A. albostriata* in the Future

**FIGURE 5:**
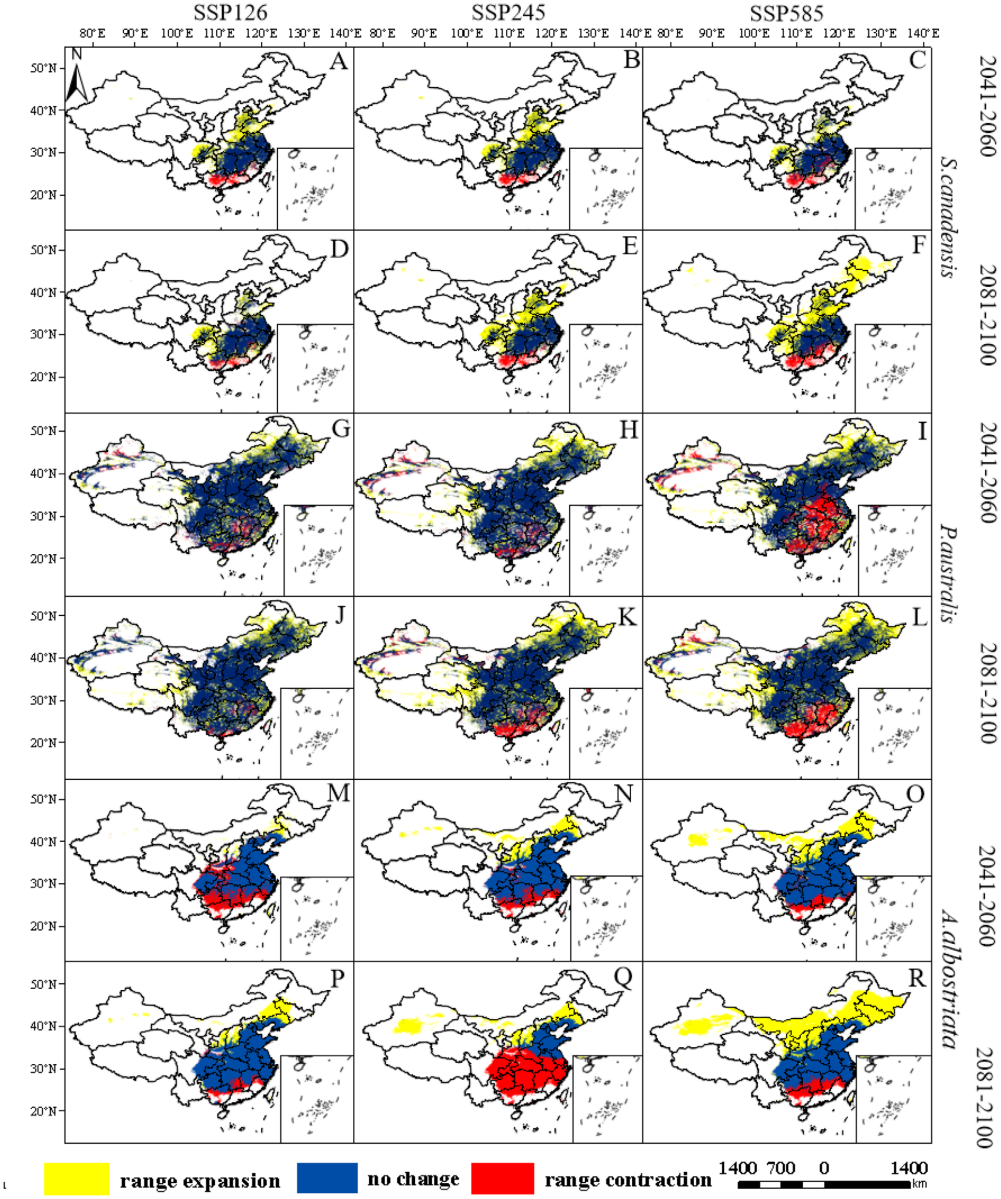
Changes in suitable habitat area for *S. canadensis* , *P. australis* , and *A. albostriata* under future climate conditions (A–C: show changes in suitable habitat area for *S. canadensis* from 2041 to 2060 under SSP126, SSP245, and SSP585 scenarios, respectively; D–F: show changes in suitable habitat area for *S. canadensis* under SSP126, SSP245, and SSP585 scenarios for 2081–2100; G–I: show changes in suitable habitat area for *P. australis* under SSP126, SSP245, and SSP585 scenarios for 2041–2060; J–L: show changes in suitable habitat area for *P. australis* from 2081 to 2100 under SSP126, SSP245, and SSP585 scenarios; M–O: show changes in suitable habitat area for *A. albostriata* from 2041 to 2060 under SSP126, SSP245, and SSP585 scenarios; P–R: show changes in suitable habitat area for *A. albostriata* from 2081 to 2100 under SSP126, SSP245, and SSP585 scenarios.)

The potential distribution area changes for the three species under current and future climate scenarios were quantified (Table 2). Compared to the current period, under the three climate scenarios for 2041–2060, the habitats of *S. canadensis* (Figure 5A–C), *P. australis* (Figure 5G–I), and *A. albostriata* (Figure 5M–O) all expanded, primarily extending around existing areas. *S. canadensis* exhibits new areas in the Xinjiang Uygur Autonomous Region under the SSP126 (Figure 5A) and SSP245 (Figure 5B) climate scenarios. Compared to SSP126 (Figure 5A) and SSP245 (Figure 5B), suitable habitat area shows a shrinking trend under SSP585 (Figure 5C). *P. australis* exhibits a shrinking trend in suitable habitat area under SSP245 (Figure 5H) and SSP585 (Figure 5I) climate scenarios (particularly in high latitudes and northern parts of its main range). *A. albostriata* showed expansion under SSP126 (Figure 5M), SSP245 (Figure 5N), and SSP585 (Figure 5O) climate scenarios (in North China and Northeast China), with its potential distribution area in the Xinjiang Uygur Autonomous Region also gradually increasing (Figure 5M–O).

**TABLE 2:** AUC and MTSS values for *Solidago canadensis*, *Phragmites australis*, and *Argyrogramma albostriata*.

Species	Period	Climate scenarios	Suitable area	Optimal area	Range expansion	No change	Range contraction
*Solidago canadensis*	Current	—	98.42	1.33	—	—	—
	2041–2060	SSP126	122.63	10.57	47.18	91.20	16.12
		SSP245	115.58	10.89	51.54	92.36	14.95
		SSP585	108.34	5.87	88.67	35.18	18.64
	2081–2100	SSP126	105.68	2.11	40.98	95.47	11.84
		SSP245	127.73	27.27	72.49	90.14	17.18
		SSP585	148.26	36.76	11.34	82.86	24.46
*Phragmites communis*	Current	—	367.38	16.07	—	—	—
	2041–2060	SSP126	408.25	54.61	82.98	364.43	20.61
		SSP245	404.55	53.62	100.33	360.34	24.70
		SSP585	384.21	60.35	107.31	304.39	80.64
	2081–2100	SSP126	407.44	58.02	100.96	373.59	11.45
		SSP245	388.95	102.02	136.11	345.72	39.32
		SSP585	353.57	138.40	150.57	329.51	55.52
*Argyrogramma albostriata*	Current	—	122.96	89.75	—	—	—
	2041–2060	SSP126	108.71	66.51	13.33	149.73	73.50
		SSP245	156.97	74.13	49.67	179.77	43.45
		SSP585	154	99	91.45	192.51	30.71
	2081–2100	SSP126	182.81	85.14	46.50	201.40	21.82
		SSP245	113.05	50.97	62.47	61.16	162.06
		SSP585	239.99	170.6	204.46	182.34	40.88

Under the three climate scenarios for 2081–2100, the total areas of *S. canadensis* (Figure 5D–F) and *A. albostriata* (Figure 5P–R) both expanded, while the total distribution area of *P. australis* (Figure 5J–L) slightly decreased. *S. canadensis* primarily expanded toward northern and northeastern China; *P. australis* potential suitable area contracted toward mid‐to‐low latitudes under all three climate scenarios; *A. albostriata* expanded its distribution range in the northeast region and the Xinjiang Uygur Autonomous Region under SSP126 and SSP585 climate scenarios. Under the SSP245 scenario, its suitable area contracted toward mid‐to‐low latitudes, but its distribution range in the Xinjiang Uygur Autonomous Region expanded.

### 3.4 Overlap Analysis of Suitable Habitats for *S. canadensis* , *P. australis* , and *A. albostriata*

**FIGURE 6:**
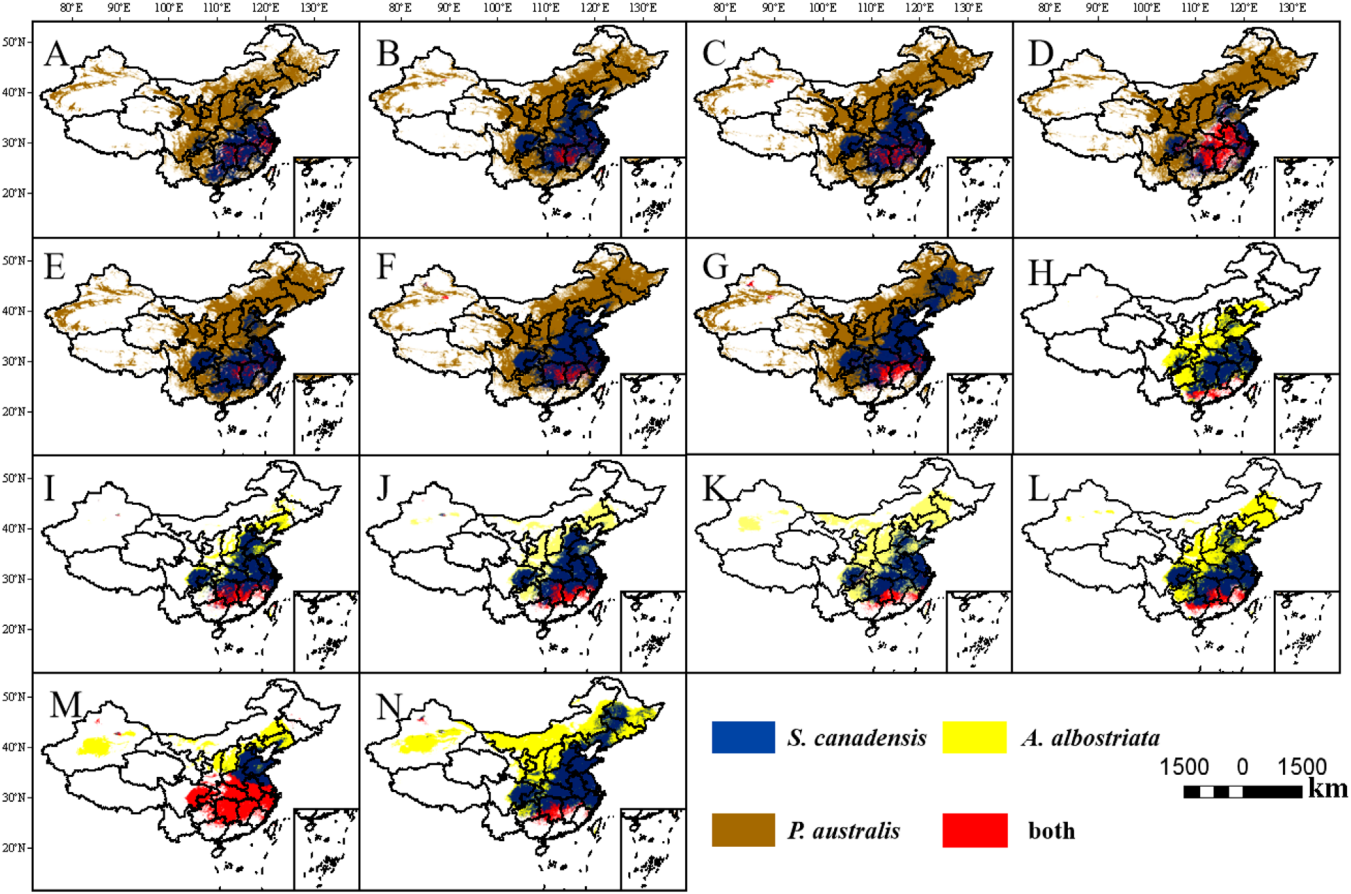
Overlapping suitable habitats of *S. canadensis* , *P. australis* , and *A. albostriata* : (B) Overlapping suitable habitat areas for *S. canadensis* and *P. australis* under the SSP126 scenario for 2041–2060; (C) Overlapping suitable habitat areas for *S. canadensis* and *P. australis* under the SSP245 scenario for 2041–2060; (D) Overlapping suitable habitat area for *S. canadensis* and *P. australis* under the SSP585 scenario for 2041–2060; (E) Overlapping suitable habitat area for *S. canadensis* and *P. australis* under the SSP126 scenario for 2081–2100; (F) Overlapping suitable habitat areas for *S. canadensis* and *P. australis* under the SSP245 scenario for 2081–2100; (G) Overlapping suitable habitat areas for *S. canadensis* and *P. australis* under the SSP585 scenario for 2081–2100; (H) Current overlapping suitable habitat areas for *S. canadensis* and *A. albostriata* ; (I) Overlapping suitable habitat area for *S. canadensis* and *A. albostriata* under the SSP126 scenario for 2041–2060; (J) Overlapping suitable habitat area for *S. canadensis* and *A. albostriata* under the SSP245 scenario for 2041–2060; (K) Overlapping suitable habitat areas for *S. canadensis* and *A. albostriata* under the SSP585 scenario for 2041–2060; (L) Overlapping suitable habitat areas for *S. canadensis* and *A. albostriata* under the SSP126 scenario for 2081–2100; (M) Overlapping suitable habitat areas for *S. canadensis* and *A. albostriata* under the SSP245 scenario for 2081–2100; (N) Overlapping suitable habitat areas for *S. canadensis* and *A. albostriata* under the SSP585 scenario for 2081–2100; Blue areas indicate suitable habitat for *S. canadensis* only; Brown areas indicate areas suitable only for *P. australis* ; Yellow areas indicate areas suitable only for *A. albostriata* ; Red areas in (A–G) indicate overlapping suitable areas for *S. canadensis* and *P. australis* ; Red areas in (H–N) indicate overlapping suitable areas for *S. canadensis* and *A. albostriata* .

The total area of overlap with *P. australis* under modern climate conditions (Figure 6A) is 14.59 × 10^4^ km^2^, primarily distributed in South Central China (parts of Hunan and Jiangxi), localized areas of South China (central Guangdong, northern Fujian), and a small portion of Taiwan Province. Under the three climate scenarios for 2041–2060, the total overlapping areas with *P. australis* were 12.52 × 10^4^ km^2^, 16.98 × 10^4^ km^2^, and 48.83 × 10^4^ km^2^, respectively. Under the SSP126 scenario (Figure 6B), the overlapping area was concentrated in eastern China and parts of central China (Hubei, Hunan, and parts of Jiangxi).; under the SSP245 scenario (Figure 6C), the overlap area slightly expanded (Hunan and parts of Jiangxi); under the SSP585 scenario (Figure 6D), the overlap area significantly expanded, covering Central China (Hunan), East China (Jiangxi), and Northern South China (Northern Guangdong, Southern Fujian). Under the three climate scenarios for 2081–2100, the total overlapping areas with *P. australis* were 8.22 × 10^4^ km^2^, 13.84 × 10^4^ km^2^, and 21.50 × 10^4^ km^2^, respectively. Under the SSP126 scenario (Figure 6E), the overlapping area was primarily concentrated in Hubei, Hunan, and parts of Jiangxi; Under the SSP245 scenario (Figure 6F), the overlap area slightly expanded but remained distributed in Hubei, Hunan, and parts of Jiangxi; under the SSP585 scenario (Figure 6G), the overlap area was primarily concentrated in Central China (Hunan), East China (Jiangxi), and South China (northern Guangdong, southern Fujian). The total overlap area between *S. canadensis* and its natural enemy *A. albostriata* under modern climate conditions (Figure 6H) is 11.80 × 10^4^ km^2^, primarily distributed in southern South China (southern Guangdong, southern Guangxi, northern Hainan) and southern Taiwan Province; Under the three climate scenarios for 2041–2060, the total overlapping areas with *A. albostriata* were 26.76 × 10^4^ km^2^, 23.74 × 10^4^ km^2^, and 18.90 × 10^4^ km^2^, respectively. Under the SSP126 scenario (Figure 6I), the overlapping area was primarily concentrated in Central China (Hunan, Jiangxi), South China (Guangdong, Guangxi). Under the SSP245 scenario (Figure 6J), the overlap area contracted toward lower latitudes. Under the SSP585 scenario (Figure 6K), the overlap area significantly decreased but remained concentrated in southern Hunan and Jiangxi, southeastern Fujian, and northern Taiwan. Under the three climate scenarios for 2081–2100, the total overlapping areas with *A. albostriata* were 20.33 × 10^4^ km^2^, 116.86 × 10^4^ km^2^, and 18.82 × 10^4^ km^2^, respectively. Under the SSP126 scenario (Figure 6L), the overlap area is primarily concentrated in southern South China (Guangdong, Guangxi, northern Hainan) and southern Central China (Hunan, Jiangxi); Under the SSP245 scenario (Figure 6M), the overlapping area significantly expanded to cover Central China (Hunan, Jiangxi, southern Hubei), South China (Guangdong, Guangxi, Fujian), and Southwest China (southern Sichuan and eastern Yunnan); Under the SSP585 scenario (Figure 6N), the overlapping area was primarily concentrated in southern Hunan and Jiangxi, northern Guangdong and Guangxi, and northern Fujian.

### 3.5 Migration of Centroid of *S. canadensis* , *P. australis* and *A. albostriata* ‐Suitable Area Under Different Climate Scenarios

**FIGURE 7:**
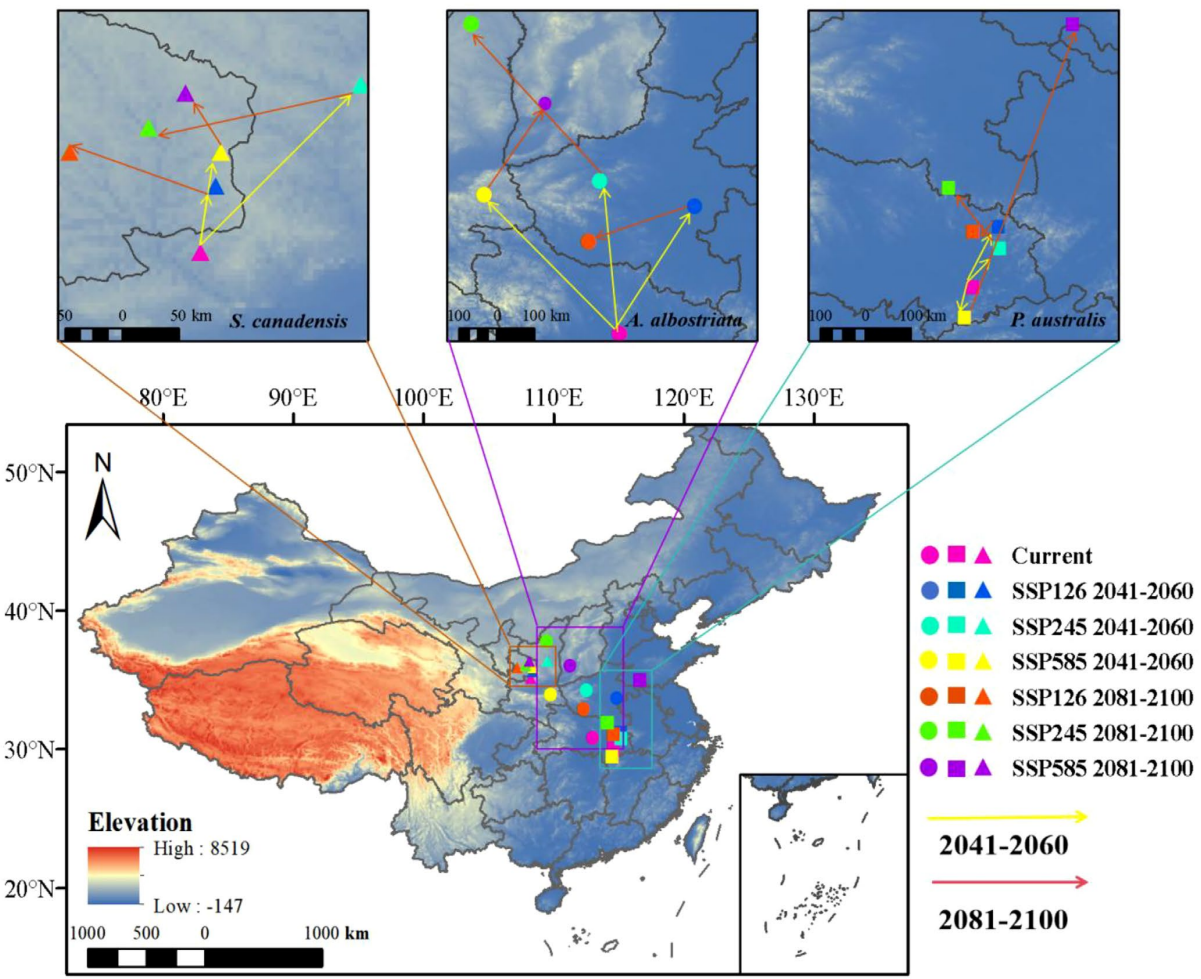
Trajectory changes in the centroid in the future for *S. canadensis* , *P. australis* , and *A. albostriata*.

Under modern climatic conditions, the centroid of *S. canadensis* is located in Xunyang County, Xianyang City, Shaanxi Province (108°17′52.800″ E, 35°6′21.600″N). The centroid of *P. australis* is situated in Daye City, Huangshi City, Hubei Province (114°37′33.600″ E, 29°59′24″ N). The centroid of *A. albostriata* is located in Jingshan City, Jingmen City, Hubei Province (113°0′36″ E, 30°49′55.200″N). During the period 2041–2060, *S. canadensis* shifted northeastward under SSP126, SSP245, and SSP585 scenarios. During 2081–2100, SSP126 and SSP585 both moved northwestward, while SSP245 shifted southwestward. For *P. australis* , during 2041–2060, SSP126 and SSP245 both shifted northeastward, while SSP585 moved southwestward. During 2081–2100, SSP126, SSP245, and SSP585 shifted southwestward, northwestward, and northeastward, respectively. For *A. albostriata* , during 2041–2060, SSP245 and SSP585 shifted northwestward while SSP126 moved northeastward. During 2081–2100, SSP126, SSP245, and SSP585 shifted southwestward, northwestward, and northeastward, respectively (Figure 7).

### 3.6 Ecological Niche Differentiation

**FIGURE 8:**
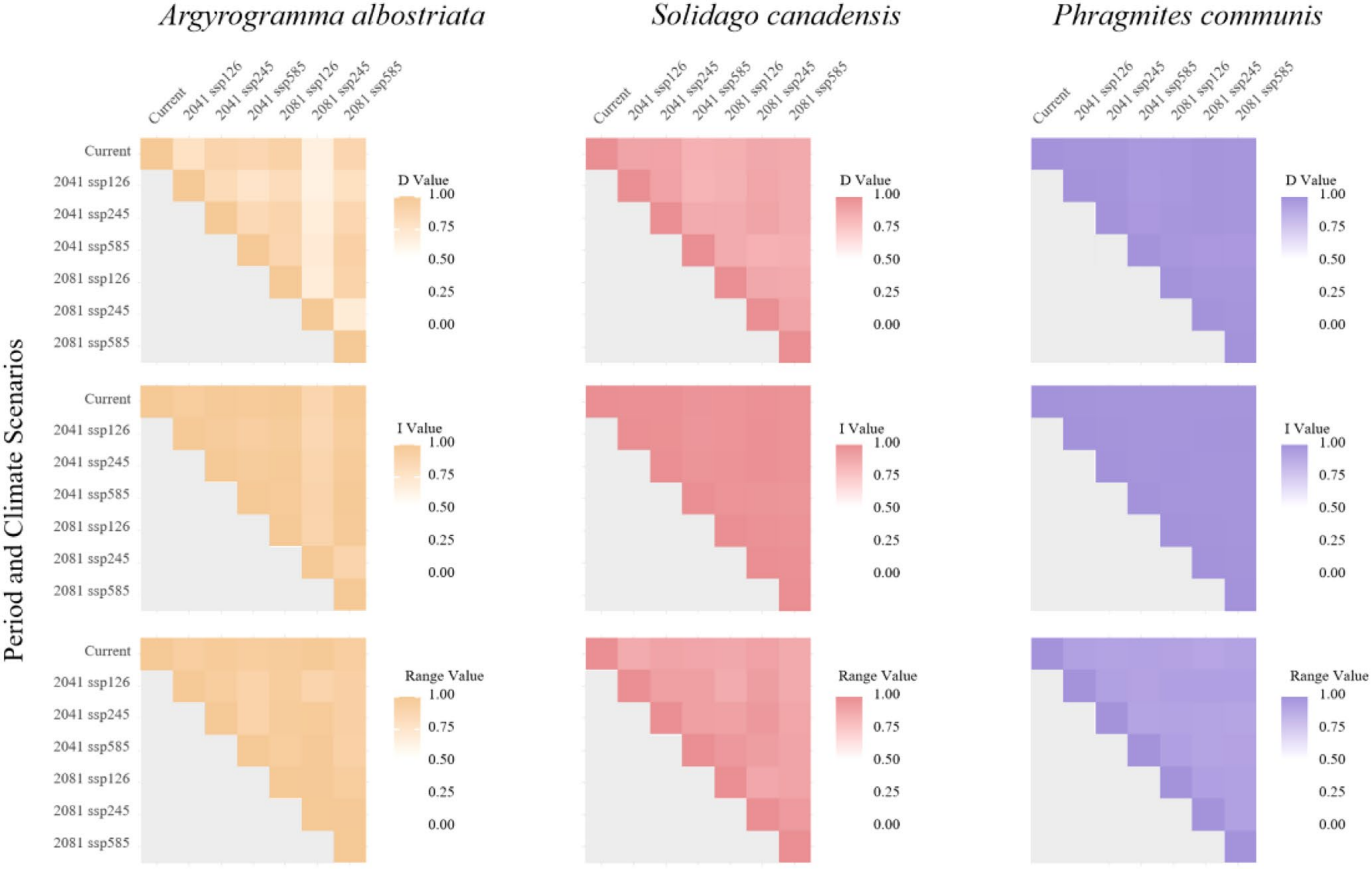
Interspecific ecological niche overlap and range overlap among *S. canadensis* , *P. australis* , and *A. albostriata*.

Using ENMTools software, we analyzed the ecological niche overlap and range overlap of *S. canadensis* , *P. australis* , and *A. albostriata* under current and near‐future (2041–2060) to future (2081–2100) climate scenarios (Figure 8). Results indicate that under current climate conditions and across all three climate scenarios (2041–2060 and 2081–2100), *S. canadensis* exhibits D‐values consistently exceeding 0.84, with I‐values exceeding 0.96. For *P. australis* , D‐values exceeded 0.90 and I‐values exceeded 0.98 under current conditions and across all three climate scenarios (2041–2060 and 2081–2100). *A. albostriata* exhibits *D*‐values exceeding 0.65 and *I*‐values exceeding 0.87 under current conditions and across the 2041–2060 and 2081–2100 climate scenarios, indicating high ecological niche overlap. Range overlap among *S. canadensis* , *P. australis* , and *A. albostriata* all exceed 0.87.

**FIGURE 9:**
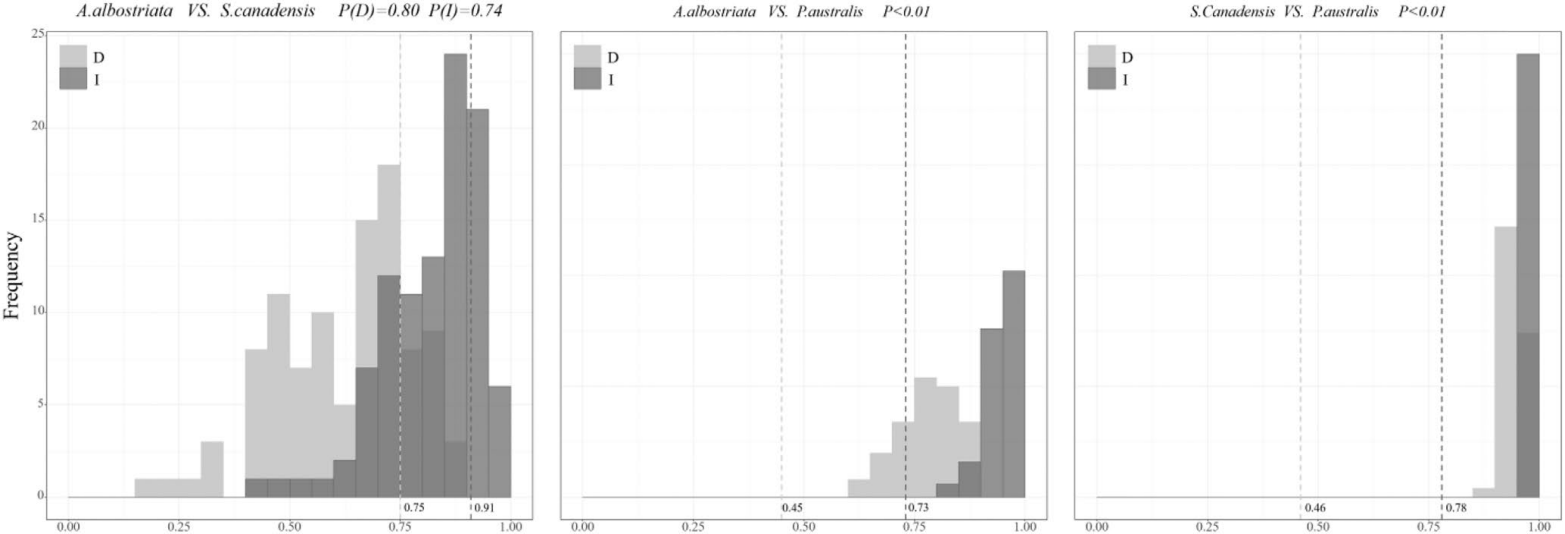
Niche identity test for *S. canadensis*, *P. australis* , and *A. albostriata* . The vertical dotted lines show the empirical values of Schoener D and Warren I, and the histograms represent the frequency of the expected Schoener D and Warren I.

Niche identity test results are presented as follows (Figure 9): Substantial niche overlap between *A. albostriata* and *S. canadensis* . The *D*‐values for this species pair were predominantly distributed between 0.00 and 0.75, with the actual values significantly higher than the expected values; substantial niche overlap between *S. canadensis* and *P. australis* The actual D‐values (0.80) was higher than the expected range (0.40–0.75), also reflecting a high degree of niche similarity; Clear niche differentiation between *A. albostriata* and *P. australis* the expected D‐values clustered between 0.60 and 0.68, which were significantly lower than the actual observed values, demonstrating clear niche differentiation between these two species.

## 4 Discussion

The model identified precipitation seasonality, mean temperature of the driest quarter, and human activity intensity as dominant drivers for *S. canadensis* distribution. This suggests that its invasion success is shaped not by a single climatic factor but by the seasonal synchronization of hydrothermal conditions coupled with anthropogenically created ecological opportunities. Human activities, by altering landscape connectivity, may provide dispersal corridors that extend beyond climatic constraints.

Under modern climatic conditions, predictions based on the MaxEnt model indicate that *S. canadensis* primarily occurs in subtropical monsoon climate zones. The model identified climate factors such as temperature and precipitation, along with human activities, as key determinants of its distribution pattern. Specifically, precipitation is a pivotal factor influencing its distribution. Specifically, precipitation is a pivotal factor influencing its distribution, which exhibits good adaptability in areas with annual rainfall of 1300–1700 mm and average annual relative humidity exceeding 70%. Crucially, the high contribution of the “Human Activity Intensity” variable in our model corresponds directly to the process of habitat fragmentation resulting from human activities (Liu et al. 2025). Activities such as road construction and water conservancy projects not only hinder the dispersal and gene exchange of native species, weakening their survival capacity, (Dos Santos et al. 2025), but may also create disturbed patches favorable for the establishment of *S. canadensis* , thereby facilitating its upslope expansion but may also create disturbed patches favorable for the establishment of *S. canadensis* , thereby facilitating its upslope expansion. This invasive species can gradually expand its distribution range by exploiting fragmented habitats and even achieve rapid transregional spread through habitat corridors. Due to frequent human activity, *S. canadensis* is expanding into higher elevations. This indicates its adaptability and reproductive capacity at higher elevations. Consequently, rising temperatures, humid environments, and intense human activity may collectively promote further invasions of *S. canadensis* within tropical and subtropical ecosystems.

Under modern climatic conditions, *P. australis* is primarily influenced by human activities, precipitation, and temperature. Warming has led to increased surface organic carbon in *P. australis* wetland soils, thereby accelerating plant growth rates (Chen et al. 2024). Secondly, changes in rainfall affect soil respiration by altering microbial metabolism through shifts in soil water potential (Li et al. 2021). Rain enhancement increases soil water potential (Gairola et al. 2019), soil microorganisms accelerate metabolic processes to balance water potential inside and outside cells, preventing cell rupture and thereby accelerating plant growth (Xu et al. 2022); *A. albostriata* is primarily regulated by temperature and rainfall. As a key ecological factor, temperature significantly impacts the insect's survival duration, population size, and geographic range by altering its metabolic rate (Skendžić et al. 2021). The temperature response curve (Figure 3I–L) indicates a unimodal relationship for *A. albostriata*, with occurrence probability increasing sharply between 10°C and 25°C, peaking around 30°C and then declining. This pattern aligns with its thermal performance curve: temperatures below 10°C likely suppress metabolism and development, while the decline beyond 30°C may approach its upper thermal limit, inducing heat stress and reducing fitness (Beermann et al. 2023). Similarly, the response of *S. canadensis* to Precipitation of the Warmest Quarter (Bio18) shows a plateau of high suitability between approximately 350 and 550 mm (Figure 3A), suggesting this range represents optimal water availability during a critical growth period, with lower values inducing drought stress and higher values potentially leading to waterlogging or disease. Additionally, variations in precipitation significantly impact their population dynamics. Ample rainfall provides a suitable moist environment conducive to adult emergence, egg hatching, and larval feeding activities, leading to explosive population growth (Fu et al. 2024). During periods of low precipitation, increased environmental dryness not only inhibits embryonic development in eggs but also leads to higher larval mortality rates, resulting in a significant reduction in population size (Yang et al. 2013). Therefore, temperature and rainfall indirectly determine whether *A. albostriata* populations can successfully colonize and expand in specific regions by influencing their physiological and ecological processes. Meanwhile, temperature, rainfall, and human activities directly affect the growth rate of *P. australis* .

However, when interpreting the relative importance of environmental variables identified by the Jackknife test, it is important to consider the potential influence of multicollinearity among predictors. Many bioclimatic variables (e.g., Bio04, Bio09, Bio10, Bio11, Bio15) are derived from the same temperature and precipitation series and thus exhibit inherent spatial correlations. Similarly, human activity intensity may correlate with certain climatic gradients. As a result, the contribution percentages of individual variables may reflect statistical redundancy rather than purely independent ecological effects. In the current study, we opted to retain a comprehensive set of environmental predictors to assess their combined influence on three biologically distinct species without a priori subjective elimination. The MaxEnt algorithm's regularization procedure and our use of 10‐fold cross‐validation help mitigate overfitting risks (Elith et al. 2011). Nevertheless, we acknowledge that the interpretation of variable importance should be approached with caution. The dominant factors identified—such as Bio18 for *S. canadensis* , HA for *P. australis* , and Bio11 for *A. albostriata* —are consistent with established ecological knowledge, supporting their biological relevance despite potential statistical interdependencies. Future studies should incorporate formal collinearity diagnostics (e.g., VIF analysis or principal component analysis) to further refine predictor selection and enhance the interpretability of variable contributions.

Global warming significantly intensifies the invasion threat posed by *S. canadensis* . The synergistic control system combining “native plant competition and specific predation regulation” holds potential for application under future climate scenarios. From the perspective of *P. australis* (Figure 6A–G), under different future emission scenarios, the overlap between the suitable habitats of *P. australis* and *S. canadensis* shows a trend of expanding toward higher latitudes and elevations with climate warming, while maintaining stability in core areas. The expansion of *P. australis* suitable habitat continuously covers the potential habitat of *S. canadensis* , which provides the necessary spatial basis for the former to exert sustained competitive pressure on the latter. The habitat range of the *A. albostriata* tracks the expansion trajectory of *S. canadensis* , defined as the synchronous and directional spatial shift of its high‐suitability areas following the expansion of its host plant's suitable range. The spatial overlap between their suitable habitats remains stable or even increases under future scenarios, providing spatial evidence for the potential sustained regulatory role of this natural enemy (Figure 6H–N). However, a crucial distinction must be made between predicted spatial overlap and actual control efficacy. Our models identify areas where the preconditions for synergistic control—namely, the co‐occurrence of competitor, enemy, and invader—are met under future climates. The realized effectiveness of this strategy, however, depends on local‐scale ecological dynamics not captured by our correlative models. As climate warming drives the expansion of *S. canadensis* habitat range, the *A. albostriata* simultaneously covers these areas. Concurrently, the extent of *S. canadensis* dispersal is significantly reduced in areas where its habitat is constrained by both the white‐banded silver moth and *P. australis* . This combined “bidirectional regulation” could potentially more comprehensively disrupts *S. canadensis* invasion chain of “dispersal‐colonization‐expansion.” Key factors determining success, such as the actual population densities of *A. alborstriata*, the intensity of resource competition from *P. australis* under specific field conditions, and potential unmodeled trophic interactions (e.g., hyperparasitoids), require empirical validation. Under high‐emission scenarios like SSP585, proactive artificial interventions (e.g., *P. australis* community restoration, targeted moth releases) can be implemented in areas with high overlap between *P. australis* and the *A. albostriata* to enhance the synergistic “competition‐feeding” effect. This combination intervention model based on native organisms also provides an ecologically sustainable paradigm for invasive species management under global change, helping to enhance ecosystem resistance and resilience to invasions.

Although climate warming drives the expansion of *S. canadensis* suitable habitat and increases invasion risks, the habitat stability of native *P. australis* and the habitat coupling of its specific predator provide geospatial feasibility for the biological control strategy of “native plant competition ‐ specific predator regulation.” Future research should prioritize monitoring the long‐term evolution of the three species' suitable ranges under medium‐to‐high emission scenarios (e.g., SSP585) to optimize control measure deployment and enhance the effectiveness of biological control against *S. canadensis* invasions under global warming.

## 5 Conclusions

Future projections further reveal that the suitable ranges of *P. australis* and *A. albostriata* can sustain persistent “competitive coverage” and demonstrate “predatory tracking” of the expanding range of *S. canadensis* . This spatial alignment offers a theoretical basis for designing a climate‐resilient synergistic biocontrol system. Several important limitations should be noted when interpreting these findings. First, model projections depend on specific future climate scenarios and assume static ecological niches, without accounting for uncertainties in climate forecasts or potential species adaptation. Second, although species distribution models identify areas of potential habitat suitability, the actual success of biocontrol relies on local‐scale biotic interactions—such as population dynamics, strength of interspecific competition, and natural enemy establishment—which are not represented in the models. Third, while ecological interpretation of environmental response curves has been refined, it remains inferential; direct physiological studies are needed to confirm the identified optimal ranges and tolerance limits. Building on these results and acknowledging the limitations, we suggest careful management actions and outline specific directions for future research. For management, resources should be prioritized in current and projected zones of high species overlap—such as wetlands in Central China and southern South China—to conduct pilot habitat‐management trials. These trials should aim to enhance *P. australis* communities and perform detailed ecological risk assessments before any intentional release of *A. albostriata* . At the same time, a dynamic monitoring system should be set up to track the spread of *S. canadensis* , especially beyond predicted overlap areas under high‐emission scenarios like SSP585. Future research should concentrate on: (1) carrying out long‐term field experiments in key overlap regions to measure the actual ecological impact of the proposed synergistic control; (2) combining population dynamics models with spatial suitability predictions to improve estimates of potential population suppression; and (3) examining whether this niche‐based “competitor–enemy” screening approach can be applied to other global invasion hotspots, such as North America and Europe. In summary, this work provides a new perspective and a planning tool for developing an ecology‐driven, spatially coordinated strategy to manage invasive species under global change.

## Author Contributions

**Jinghui Zhang:** data curation (equal), writing – original draft (equal), writing – review and editing (equal). **Xiaoying Xiao:** data curation (equal). **Wei Huang:** data curation (equal), software (equal). **Yuxin Huo:** software (equal), visualization (equal). **Yuxin Zhang:** methodology (equal), visualization (equal). **Shujian Zhang:** software (equal), validation (equal). **Xinyi Huang:** methodology (equal), resources (equal). **Muhammad Umair Hassan:** software (equal), validation (equal). **Yuxi Xue:** project administration (equal), software (equal). **Qitao Su:** funding acquisition (equal), project administration (equal), writing – review and editing (equal). **Yian Xiao:** formal analysis (equal), funding acquisition (equal), writing – review and editing (equal).

## Funding

This work was supported by the National Natural Science Foundation of China (42467035 and 41561012), the Key Laboratory of Jiangxi Province for Biological Invasion and Biosecurity (2023SSY02111), and the Jinggangshan University Doctoral Research Initiation Project (JZB2523).

## Conflicts of Interest

The authors declare no conflicts of interest.

## Supporting information


**Table S1:** The 23 environmental variables used for model prediction.

## Data Availability

All data and code supporting this study are deposited in publicly accessible repositories to ensure reproducibility. Occurrence records of *Solidago canadensis* , *Phragmites australis* and *Argyrogramma albostriata* are sourced from multiple databases: the Global Biodiversity Information Facility (GBIF; https://www.gbif.org/), the Chinese Virtual Herbarium (CVH; https://www.cvh.ac.cn/), the China Animal Scientific Database (http://www.zoology.csdb.cn), the Encyclopedia of Life (https://www.eol.org/zh‐CN), and published records from the Flora of China. Bioclimatic variables are retrieved from WorldClim version 2.1 (https://www.worldclim.org/).
