# Supplementary material for: Phragmites australis and Argyrogramma albostriata Suppress the Invasion of Solidago canadensis in China Under Future Climate Change
Source: Ecol Evol. 2026 Apr 22;16(4):e73573. doi: 10.1002/ece3.73573 (PMC13102494; doi:10.1002/ece3.73573)
Supplement: Supplementary file 1 — Table S1: The 23 environmental variables used for model prediction. [file ECE3-16-e73573-s001.docx]

**Suppl. Table 1** The 23 environmental variables used for model prediction.

| Variables | Abbreviations |
| --- | --- |
| Annual mean temperature  Mean diurnal range  Isothermality | Bio01  Bio02  Bio03 |
| Temperature seasonality | Bio04 |
| Max. temperature of warmest month | Bio05 |
| Min. temperature of coldest month | Bio06 |
| Temperature annual range | Bio07 |
| Mean temperature of wettest quarter | Bio08 |
| Mean temperature of driest quarter | Bio09 |
| Mean temperature of warmest quarter | Bio10 |
| Mean temperature of coldest quarter  Annual precipitation | Bio11  Bio12 |
| Precipitation of wettest month  Precipitation of driest month  Precipitation seasonality | Bio13  Bio14  Bio15 |
| Precipitation of wettest quarter  Precipitation of driest quarter  Precipitation of warmest quarter  Precipitation of coldest quarter | Bio16  Bio17  Bio18  Bio19 |
| Elevation | elev |
| Human activity | HA |
| Slope | slope |
| Aspect | aspect |
